# The Effect of Diet Quality and Wing Morph on Male and Female Reproductive Investment in a Nuptial Feeding Ground Cricket

**DOI:** 10.1371/journal.pone.0003437

**Published:** 2008-10-17

**Authors:** Matthew D. Hall, Luc F. Bussière, Robert Brooks

**Affiliations:** 1 Evolution & Ecology Research Centre and School of Biological, Earth & Environmental Sciences, University of New South Wales, Sydney, New South Wales, Australia; 2 School of Biological & Environmental Sciences, University of Stirling, Stirling, Scotland, United Kingdom; University of Exeter, United Kingdom

## Abstract

A common approach in the study of life-history trade-off evolution is to manipulate the nutrient content of diets during the life of an individual in order observe how the acquisition of resources influences the relationship between reproduction, lifespan and other life-history parameters such as dispersal. Here, we manipulate the quality of diet that replicate laboratory populations received as a thorough test of how diet quality influences the life-history trade-offs associated with reproductive investment in a nuptial feeding Australian ground cricket (*Pteronemobius sp.*). In this species, both males and females make significant contributions to the production of offspring, as males provide a nuptial gift by allowing females to chew on a modified tibial spur during copulation and feed directing on their haemolymph. Individuals also have two distinct wing morphs, a short-winged flightless morph and a long-winged morph that has the ability to disperse. By manipulating the quality of diet over seven generations, we found that the reproductive investment of males and females were affected differently by the diet quality treatment and wing morph of the individual. We discuss the broader implications of these findings including the differences in how males and females balance current and future reproductive effort in nuptial feeding insects, the changing nature of sexual selection when diets vary, and how the life-history trade-offs associated with the ability to disperse are expected to differ among populations.

## Introduction

Understanding the relationship between reproductive investment and other functionally important traits is the central ongoing challenge for life-history theory [1], [2]. Empirical research on this subject is particularly challenging because individuals vary in both the amount of resources they can obtain (acquisition) and how they invest those resources in specific traits (allocation) and together these two levels of variation can produce complex outcomes [3]–[6]. To gain a complete and integrated view of life-history trade-offs, our understanding of how individuals allocate resources to reproduction and other important life-history traits needs to incorporate appropriate manipulations or measures of resource acquisition [7]. Accordingly, studies that vary the quality or quantity of diet that individuals receive and measure the immediate phenotypic responses can tell us a great deal about the nature of reproductive investment and trade-offs between traits.

Diet manipulation has commonly been used to understand the relationship between reproductive investment and aging [8]–[11], the influence of condition on female preference and male sexual advertisement [12]–[14] and the nature of sexual dimorphism [15], [16]. Although useful in understanding the nature of life-history trade-offs, the immediate response to dietary manipulation, however, does not necessarily indicate how species adapt to changes in their nutritional environment. In *Plutella xylostella*, for example, Warbrick-Smith et al [17] demonstrated that the laboratory populations of caterpillars that were restricted to high-carbohydrate diets for multiple generations evolved the ability to reduce fat storage, whereas the initial response to those diets typically results in excess lipid storage and reduced fitness. Their study highlights the usefulness of manipulating the nutritional environment for multiple generations as a thorough examination of the adaptive responses in allocation that result from changes in the availability of resources.

Here we present the first results from a large laboratory experiment in which we kept replicate experimental populations of a nuptial-feeding Australian ground cricket (*Pteronemobius sp.*) on high- or low-quality diets for seven generations. In the genus *Pteronemobius*, the males provide a nuptial gift by allowing females to chew on a modified tibial spur and feed directly on their haemolymph during copulation [18]. To entice females to mate, a male will engage in a courtship call and dance that, if successful, ends with the female briefly mounting him in a mock copulation. The male then prepares a spermatophore before the female mounts him for a true copulation in which their genitalia are engaged and the male's hind leg is brought forward to facilitate spur chewing. In a similar nuptial feeding ground cricket, *Allonemobius socius*, the duration of spur chewing relates directly to the size of the gift [19] and the degree of material benefits received [20]. As for other Orthoptera with nuptial gifts [21]–[23], body size is an important component of the mating system as females tend to prefer larger males due to the greater sized gifts they can provide [24].

Nuptial feeding insects are particularly suited to studying male and female allocation of resources towards reproduction and other key aspects of life-history, such as lifespan and dispersal. First, both males and females invest substantially in individual mating episodes, and thus must closely attend to the costs and benefits of mating under different environmental circumstances. Second, the economics of nuptial feeding mating systems are potentially easy to manipulate by varying the quality of resources available. In the bush cricket *Kawanaphila nartee*, for example, both males and females contribute a large proportion of their energetic intake towards reproduction via spermatophore and egg production respectively [25]. However, because the per mating reproductive energy expenditure of each sex varies depending on the quality of nutrient resources available [26], [27], resource limitation results in greater energy expenditure and mate choice by males and causes females to compete for access to males and their relatively more valuable nuptial gifts [28]. A similar situation prevails in a variety other nuptial feeding insects [29], [30]. Last, we can quantify investment in dispersal as many insect species [reviewed in 31], feature individuals of two distinct wing morphs: a micropterous (short-winged) morph that lacks the ability to fly; and a macropterous (long-winged) morph that has a substantial potential to disperse. Comparisons of trait values across the morphs can indicate the trade-offs associated with macroptery and importantly the costs of dispersal.

Based on theoretical models of life-history trade-offs [32], we can make clear predictions about how the relationships between the life-history traits of *Pteronemobius* should differ across the experimental populations of each treatment. Under conditions where the genetic variance-covariance relationships between traits do not change substantially, such as in this study where the populations were allowed to adapt to the diet treatments for only seven generations, the regression equation describing a trade-off is predicted to change in its intercept but not its slope [33]. Such predictions are clearly supported by the nature of the geographic variation in the trade-off function between female fecundity or male call duration and flight capability (measured as DLM mass) for the sand cricket *Gryllus firmus* [33], [34]. Moreover, the variation in the trade-off across multiple wild populations indicates that the trade-off is not a fixed function and highlights the dynamic nature of life-history trade-off evolution in general. In this study, therefore, we should expect that phenotypic trade-offs will naturally vary among the different experimental populations, but the variation will be more likely in mean trait values rather than the correlations between traits involved in the trade-off.

Using crickets from the experimental populations, we assessed the influence of the diet treatment on the relationships between wing morph, sex-specific reproductive investment and lifespan. This provided insights into how male and female reproductive investment responds to variation in resource quality, the costs associated with the ability to disperse and whether the costs to dispersal vary with diet quality. Finally, using regression analysis, we also examined if the diet treatments or the wing morph types influenced the trade-offs between components of male and female fitness, such as male gift size and female fecundity, and other important life-history traits. Specifically we tested the theoretical life-history prediction that the nature of specific trade-offs will remain the same across the different treatments even if average trait values change. Together our findings have important implications for the influence of diet quality on how males and females balance current and future reproductive investment, the dynamics of sexual selection when environments vary, and the nature of sex-specific life-history trade-offs associated with dispersal in the Australian ground cricket, *Pteronemobius sp.*

## Methods

We studied an undescribed Australian ground cricket from the genus *Pteronemobius* (Family: Gryllidae). The crickets were originally collected in April 2003 from Waramanga (35°21′S, 149°03′E) in the Australian Capital Territory, Australia. This species is catalogued in the Australian National Insect Collection where voucher specimens were deposited in July 2006. Initially, we collected approximately 60 crickets of each sex and maintained them in the laboratory at densities of less than 200 animals in each of four large containers for two generations before establishing the experimental populations. This reduces the likelihood that any field-based environmental or maternal effects would influence the trajectories of the different populations [35]. We then randomly assigned newly hatched nymphs from all of these containers to five replicate populations on each of two diets of differing quality (see below), and maintained these populations through seven discrete generations.

### Experimental populations

We established two different diet treatments by manipulating the protein content of food provided to crickets throughout their life. The two diets both consisted of powdered high-protein fish-rearing pellets (Nova Lo: 50% protein, Skretting Australia, TAS, Australia) mixed with oatmeal (Quick Oats: 11% protein, Farmland, VIC, Australia): the high quality diet was a blended dry weight mixture of 75% fish rearing pellets to oatmeal, while the low quality diet was a mixture of 25% fish-rearing pellets to oatmeal. Preliminary data from the generation used to establish the populations showed that for both males (*t*-test: *t_125_* = 4.950, *p*<0.001) and females (*t*-test: *t_124_* = 2.422, *p* = 0.017) individuals reared on the high quality diet (average weight (mg)±SE: males = 10.620±0.166, females = 15.160±1.018) were significantly heavier at eclosion than those reared on the low quality diet (average weight (mg)±SE: males = 9.318±0.204, females = 12.413±0.287). Similar diets have been used successfully in previous manipulative studies of the effect of diet quality on male sexual display and female condition in the Australian black field cricket, *Teleogryllus commodus* [10], [12].

For each of the two diet quality treatments we established five replicate experimental populations in the laboratory. To start each experimental population, 400 newly hatched nymphs were housed in a large plastic container (55×40×22 cm) provided with the appropriate diet *ad libitum*, egg cartons for shelter and dampened cotton wool in a Petri dish as both a source of water and for oviposition. The food and egg pads were replaced weekly and the egg pads stored in labelled plastic containers (16×11×7 cm) once adults were present in the population. From the egg pads, newly hatched nymphs were collected weekly and used to establish a new generation according to their diet quality treatment and replicate population identity (i.e., there was no overlapping of generations). To control for differences in rearing density, only 75 nymphs in total each week per population contributed to the next generation. The 10 populations were kept in a controlled temperature room (28°C, 14:10 hour light: dark cycle) and maintained in this way for seven generations.

In the final generation we removed and isolated late instar nymphs individually in small plastic containers (8×8×5 cm). A total of 20 males and 20 females were removed from each experimental population and maintained on their treatment diets *ad libitum*, with vials of water stoppered with cotton wool and a piece of egg carton for shelter. Every day we checked for eclosions, weighed the new adults and then measured the length and width of their pronotum. From these three measures we extracted a single principal component that described 80 percent of the variation in the three body size measures. At eclosion we also recorded the wing morph (micropterous or macropterous) for each individual. Throughout their life the crickets were kept in a controlled temperature room and monitored for survival on a daily basis.

### Quantifying reproductive investment

To quantify male reproductive investment we video-recorded mating trials in order to estimate the size of the gift provided to females, the number of matings occurring in a 12 hour period and the attractiveness of the male. Trials were conducted by randomly pairing males and females from the same experimental populations and filming the mating pair using a video camera equipped with infrared sensing and a built-in infrared source (model DCR-TRV340E, Sony Corporation, Tokyo, Japan). The camera was attached to a computer running time-lapse computer recording software (RBCap, www.rbartick.com/rbcap/), which captured individual frames every 15 seconds instead of continuous recording, allowing for more manageable data collection. Each mating pair was placed in a small plastic container (7×7×5 cm) with dampened paper towel lining the base. The plastic containers were randomly arranged into four rows of three and placed under the tripod mounted camera. The trials ran for twelve hours, beginning at six in the evening. All crickets used in the behavioural trials were between 7 and 14 days old, post-eclosion.

We scored the number and duration of mating events for a given male and female pair by counting the number of frames for which mounting was observed and converting this to seconds. We classified a mock copulation as any time a female mounted a male for less than 1 minute (4 frames), although mock copulations typically last for 30 seconds (pers. obs.). We estimated a male's attractiveness as the inverse of the time taken from the start of the trial until the first mock copulation. We recorded the number of copulations and the average duration of the true copulations. In tibial spur feeding crickets, such as *Allonemobius socius*, the length of copula duration is used as an estimate of gift size [24], as this duration is highly correlated with the amount of haemolymph lost by the male [19]. Accordingly, we used the mean copulation duration as the estimate of a male's mean nuptial gift size.

Following the mating trials, we quantified female reproductive investment by characterising aspects of female fecundity. Females were provided with a small vial of water stoppered with cheesecloth for both water and oviposition. After one week the vials were removed and replaced with new vials for a second week. Once we removed a vial, the cheesecloth was unrolled and the total number of eggs counted. A random section of cheesecloth containing 50 eggs was then removed and placed in an individually labelled plastic container (5×5×5 cm), lined with dampened paper towel. The containers were monitored daily and any hatched nymphs counted and removed. From the remaining eggs (not isolated for hatching success measures), the width and length of five randomly selected eggs were measured using the graduated eyepiece of a dissecting microscope (Leica MS 5, Leica Microsystems, NSW, Australia). A single principal component describing egg size was extracted from these two measures. For a given female, therefore, we recorded the total number of eggs laid over the two week period, the average hatching success and the average egg size.

### Changes in the frequency of wing morph types

To test whether the frequency of the two wing morphs was influenced by diet, sex or an interaction between these factors, we used a three-way contingency table analysis [36 pp 743–6]. We first obtained the observed frequencies of the two types of wing morph split by diet quality treatment and sex. We then used Chi-square statistics to test for overall independence of wing morph on diet and sex simultaneously and then partitioned this effect into diet and sex effects and an interaction between diet and sex.

### The effect of diet quality and wing morph

We assessed the effect of diet quality and wing morph on life-history traits using a mixed model ANOVA, where diet treatment and wing morph type were fixed factors and experimental population nested within treatment was a random factor [as per 37]. The treatment by wing morph interaction term assessed whether the relationship between wing morph and the traits of interest remained the same in both the diet environments. Before analysis, however, male attractiveness was transformed using natural logarithms and average mating duration was transformed using the square root transformation as both were positively skewed. The transformations resulted in the residuals from the appropriate mixed model ANOVA having a normal distribution. All analyses were conducted in JMP (version 7: SAS Institute Inc, NC, USA).

In addition to testing whether diet quality and wing morph influenced mean trait values, we used multiple regression to determine whether the relationships between reproductive investment and other important life-history traits varied with diet quality and wing morph. For males we calculated overall mating investment by multiplying the number of matings by the average mating duration. We then estimated the relationship between overall mating investment and male body size, male attractiveness and male longevity using a sequential model-building approach [38] with treatment as a fixed factor and experimental population modelled as a random effect nested within treatment. First, we assessed if the overall relationship followed a linear or non-linear trend by using partial *F*-tests to statistically test if the addition of the correlational and quadratic terms improved the fit of the regression model containing only linear terms. We then used the resulting model to assess if the relationship between male mating investment and the other traits of interest differed significantly between the different diet treatments and wing morph types by assessing separately if the treatment or wing morph interaction terms improved the fit of the regression model. We then repeated the process for females. First, we calculated predicted fecundity based on our estimates of the number of eggs laid and average hatching success. We then estimated the relationship between predicted fecundity and female body size, egg size and female longevity following the same stepwise process as for males. One again, using partial *F*-tests we tested for differences in the relationship between the traits with respects to diet treatment and wing morph.

## Results

To assess the general effects of the diet treatment, we quantified both the changes in male and female weight at eclosion and the changes in the frequency of micropterous and macropterous individuals. We found that the diet treatment significantly altered the weight of crickets at eclosion. For both males (*F*_1, 8_ = 7.593, *p* = 0.025) and females (*F*_1, 8_ = 10.090, *p* = 0.013) individuals from the high quality diet populations (average weight (mg)±SE: males = 11.029±0.372, females = 13.262±0.380) were significantly lighter than those from the low quality diet populations (average weight (mg)±SE: males = 12.073±0.118, females = 14.732±0.297). However, the diet treatment did not alter the frequency of the two wing morphs (Table 1). A three-way contingency table revealed that wing morph frequency nevertheless depended on diet and sex (overall significance: χ^2^ = 51.367, df = 4, *p*<0.001). Partitioning of this table revealed that the frequencies of micropterous and macropterous individuals did not differ between the two diet treatments (χ^2^ = 0.251, df = 1, *p* = 0.616). However, males showed an excess of micropterous individuals, while females had an excess of macropterous individuals (χ^2^ = 50.624, df = 1, *p*<0.001). There was no significant interaction between the frequency of the wing morphs across sex and diet quality treatments (interaction: χ^2^ = 0.492, df = 1, *p* = 0.483).

**Table 1:** The observed frequencies for the two types of wing morph partitioned by diet quality treatment and sex.

	Micropterous	Macropterous
**By diet treatment**		
High quality	109	91
Low quality	104	96
**By sex**		
Male	142	58
Female	71	129

In total, 20 newly eclosed adult males and females were randomly sampled from each experimental population, yielding 100 males and 100 females per treatment.

### Male reproductive investment

**Figure 1:**
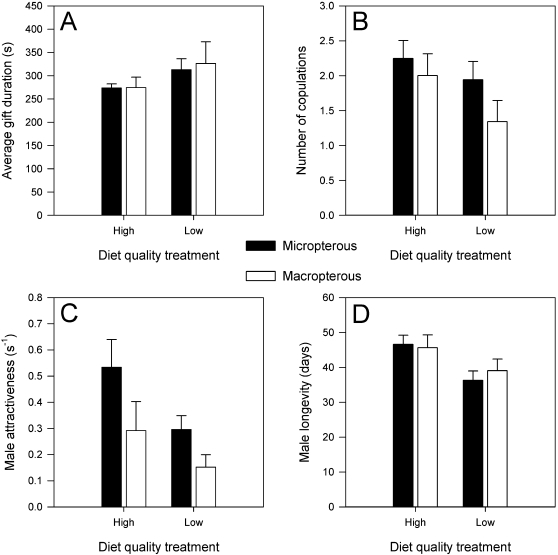
The effect of diet quality treatment and wing morph type on male reproductive investment. The means and standard errors for the diet treatment and wing morph factors are calculated based on the average values for each of the experimental populations.

The results of the mixed model analyses of the effects of diet treatment and wing morph versus male reproductive investment are shown in Table 2, while the means are shown in Figure 1. Although it appears that males from the low quality diet treatments provided larger nuptial gifts (Figure 1A) on fewer occasions (Figure 1B), both the estimates of male reproductive investment did not vary significantly between the diet treatments. Male attractiveness and male lifespan, however, did vary between the two diet treatments. Males from the high quality diet populations were marginally more attractive (Figure 1C) and lived approximately 10 days longer (Figure 1D) than males from the low quality diet populations. Likewise, only the number of copulations and male attractiveness differed significantly between the two wing types. In general, micropterous individuals obtained a greater number of copulations and were more attractive than macropterous individuals. The type of wing morph, however, had no effect on the average nuptial gift size provided to females or on male longevity. For all traits measured, the treatment by wing morph interactions were not significant, indicating that the effect of wing morph on the number of copulations and male attractiveness was consistent across the experimental populations of both diet quality treatments.

**Table 2:** The mixed model analyses for the effects of diet quality treatment and wing morph type on estimates of male and female reproductive investment.

Trait	Diet quality treatment			Wing morph type			Interaction		
	*F*-ratio	df	*p*	*F*-ratio	df	*p*	*F*-ratio	df	*p*
**Male reproductive investment**									
Average gift size	1.254	1, 10.8	0.287	0.017	1, 134	0.897	0.170	1, 134	0.681
No. Copulations	1.788	1, 8.7	0.215	5.770	1, 183	0.017	1.028	1, 183	0.312
Attractiveness	4.527	1, 9.2	0.062	11.650	1, 157	<0.001	0.242	1, 157	0.624
Male lifespan	5.392	1, 9.5	0.044	0.123	1, 195	0.727	0.530	1, 195	0.468
**Female reproductive investment**									
Egg number	6.778	1, 8.4	0.030	1.663	1, 195	0.199	0.408	1, 195	0.524
Hatching success	7.492	1, 8.1	0.025	2.326	1, 172	0.129	0.230	1, 172	0.632
Egg size	5.567	1, 8.3	0.045	0.520	1, 170	0.472	0.470	1, 170	0.494
Female lifespan	0.059	1, 8.6	0.815	0.806	1, 193	0.370	0.518	1, 193	0.472

The model analysed included diet quality treatment and wing morph type as fixed effects, and experimental evolution population nested within treatment as a random effect.

Using regression analysis, we assessed the relationship between overall male reproductive investment, as summarised by the total copulation duration, and other male characteristics such as body size, attractiveness and longevity. Partial *F*-tests showed that the relationship followed a significant linear trend (*F*_3, 165_ = 3.771, *p* = 0.012), as the addition of the correlational and quadratic terms did not improve the overall fit of the model (*F*_6, 159_ = 0.852, *p* = 0.532). Specifically, we found that total copulation duration increased with both male size (***β*** = 0.095, *p* = 0.020) and male attractiveness (***β*** = 0.070, *p* = 0.030) but there was no significant relationship with male lifespan (***β*** = 0.040, *p* = 0.222). These relationships did not differ significantly between the diet treatments or between wing morphs as the addition of the linear coefficients by treatment (*F*_3, 162_ = 0.851, *p* = 0.468) or wing morph (*F*_3, 162_ = 1.003, *p* = 0.393) interactions did not improved the fit of the linear coefficients only model.

### Female reproductive investment

**Figure 2:**
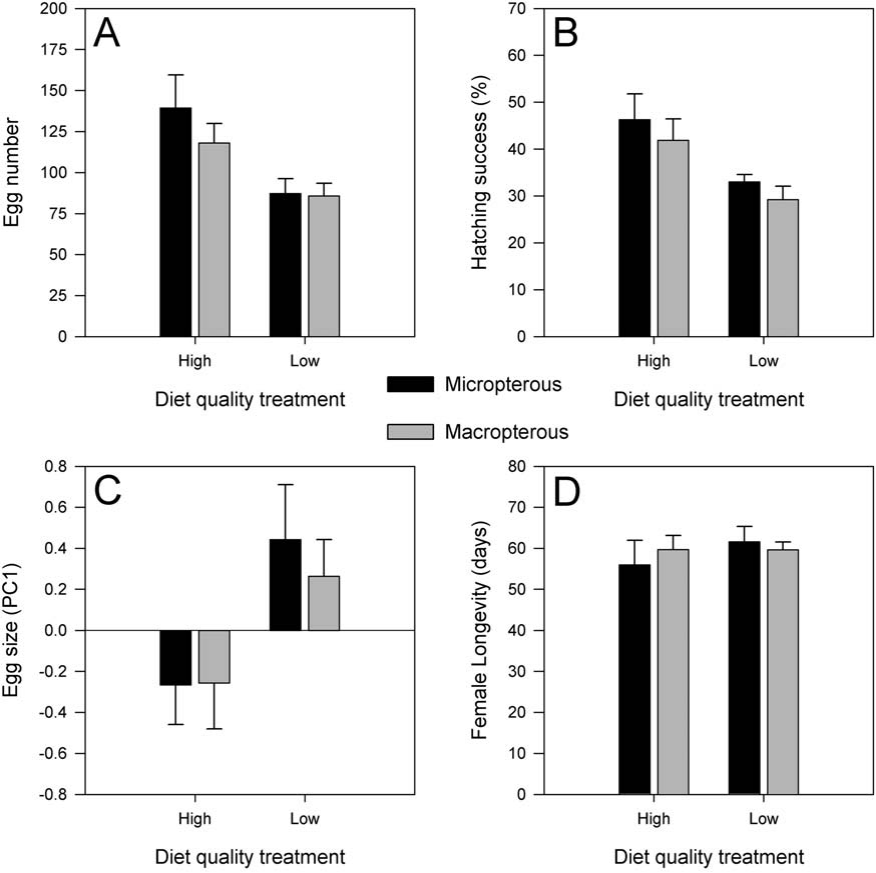
The effect of diet quality treatment and wing morph type on female reproductive investment. The means and standard errors for the diet treatment and wing morph factors are calculated based on the average values for each of the experimental populations.

The results of the mixed model analyses of female reproductive investment are shown in Table 2, while the means are shown in Figure 2. We found that aspects of female reproductive investment differed significantly between the different diet quality treatments. Females from the high quality diet populations laid almost 45 percent more eggs in two weeks (Figure 2A) and had greater than 10 percent higher hatching success (Figure 2B) than females from the low quality diet populations. Females from the low quality populations, however, laid significantly larger eggs (Figure 2C). Moreover, there was no significant difference between the two treatments in female lifespan, as all females lived for approximately 60 days post eclosion (Figure 2D). For all traits measured there were no significant differences between the micropterous and macropterous individuals and there were no significant treatment by wing morph interactions.

Once again, we used regression analysis to assess the relationship between overall female reproductive investment, estimated as predicted fecundity, and other female traits including body size, egg size and longevity. The partial *F*-tests indicated that that the relationships followed a significant linear trend (*F*_3, 162_ = 2.862, *p* = 0.039), as the addition of the correlational and quadratic terms did not improve the overall fit of the model (*F*_6, 156_ = 0.661, *p* = 0.681). We found that predicted fecundity increased with female lifespan (***β*** = 0.129, *p* = 0.0216), but there was no significant relationship with female size (***β*** = −0.046, *p* = 0.424) or egg size (***β*** = 0.057, p = 0.345). As per the analysis for male reproductive investment, we found that the relationship between predicted fecundity and female lifespan, body size and egg size did not differ between the different diet quality treatments (*F*_3, 159_ = 0.891, *p* = 0.447) nor between individuals with different wing morphs (*F*_3, 159_ = 0.718, *p* = 0.542).

## Discussion

By manipulating the quality of diet received by replicate laboratory populations of the Australian ground cricket *Pteronemobius sp.*, we found significant differences in how males and females allocate resources towards reproduction, longevity and dispersal. Our results indicate that the reproductive investment of males and females are differentially affected by both the diet quality treatment and wing morph of the individual. Moreover, both males and females were significantly heavier on the low quality diet treatment after seven generations whereas the opposite pattern was observed when we initially established the populations. Accordingly, although we are unable as part of this study to separate the immediate effects of dietary intake from the adaptation that may have occurred over the seven generations to the different diets, our results nevertheless suggest that the observed differences may represent more than just a functional dietary response. This phenotypic divergence between the treatments has important implications for how males and females balance current and future reproductive effort when faced with resource challenges, the changing nature of sexual selection across different environments, and the costs associated with the ability to disperse.

### Reproductive investment and the costs of mate choice

Our findings show that males derived from populations that have experienced low quality diets maintained their current reproductive investment in terms of average gift size and the number of matings but were marginally less attractive than high quality diet males. In *Pteronemobius sp*, therefore, it appears that male nuptial gifts are not dependant on resource availability unlike many other nuptial feeding insect species where the size of the gift provided depends on the amount of food available or the quality of the diet [39], [40]. A similar pattern to the one that we observed has been documented in the bushcricket *Requena verticalis*, whereby males maintain investment in nuptial gifts despite resource limitation [41], [42] but reduce the amount of energy allocated to mate attraction [43]. In contrast with males, however, female reproductive investment appears to depend on the quality of the resources available. Females from low quality diet populations laid fewer, larger sized eggs and had lower hatching success. These effects are due to the decrease in diet quality and not to reduced nuptial gift size because males maintain their gift size across diets. It remains possible, however, that changes in nuptial gift quality [44], which we were unable to detect as part of this study, may contribute to the reduction in female fecundity and hatching success.

How males and females allocate resources towards reproduction has important consequences for the nature of sexual selection and the strength of the conflict between the sexes. Theoretical studies suggest that the relative costs of breeding to males and females, in terms of reduced future reproductive potential or increased mortality, will influence the relative strengths of mate choice in the sexes [45]–[47]. Accordingly, sex-specific investment in reproduction when faced with resource challenges and the resulting influence on future reproductive potential via reduced lifespan can be used to predict the nature and direction of sexual selection in a mating system. In the Australian bushcricket *Kawanaphila nartee*, for example, where the sex-roles can be reversed by resource manipulation [28], the costs of breeding were significantly higher for males when resources were limited [48]. Similarly, our results suggest that male and female *Pteronemobius* differ in how they balance current and future reproduction in poor quality environments. Females on the poor quality diet, for example, appear to reduce their current reproductive effort but retain a similar longevity relative to females on the high quality diet. For males, however, the maintenance of current reproductive investment in the low quality diet populations may have come at a significant cost in the form of a shorter adult lifespan. We predict that the different ways in which males and females respond to diet quality should make females less choosy and males more so on low quality diets than on high quality diets, particularly as the gifts that males provide make them a valuable resource for females when conditions are poor.

An obvious explanation for the reduced male lifespan on the poor quality diet is that the greater costs of providing the same sized nuptial gift in a poor quality environment are manifested as the reduced allocation of resources towards somatic maintenance and repair [49]. It is also possible that females from the low quality diet populations impose greater costs for a given duration of feeding than females from the high quality diet populations. Finally, the makeup of the diet itself may have contributed to the poorer survival. Excess carbohydrate intake, for example, has been shown to cause increased mortality in the caterpillar, *Spodoptera littoralis* [50], although high carbohydrate, low protein diets have been shown to maximise longevity in the more closely related field cricket *Teleogryllus commodus* [51]. These possible causes are not mutually exclusive so it is possible that changes in the allocation of resources, the direct costs imposed by females and the physiological costs of a low protein or high carbohydrate diet all contributed to the poorer survival of males from the low quality diet populations. By not mating males and female from each treatment to a random control stock, however, we are unable to dissect the sex-specific differences in how male and female behaviours contribute to the costs of mating for males.

### Population differences in life-history trade-offs

The wing dimorphisms of many insect species are commonly used to study physiological and evolutionary consequences of the trade-off between reproduction and flight capability [31], [52]–[54]. For both males and females, the trade-off is believed to be mediated by differences in energetic stores, with the micropterous form reducing investment in flight muscle maintenance and flight fuels [55], [56]. In *Pteronemobius sp.*, however, only males appear to be significantly influenced by the trade-off between dispersal ability and reproductive effort. Macropterous males, for example, were less attractive and obtained fewer matings than micropterous rivals. In contrast, aspects of female fecundity, such as hatching success and egg number were not phenotypically associated with the morph of the female. The lack of a negative relationship between investment in dispersal and reproduction for females is surprising given that previous studies on other species of crickets have typically demonstrated that the flightless morph typically has increased reproductive output [31], [52], [57]. Nevertheless, the differences between the sexes in the fitness costs of macropterous wing morph may explain why, in general, males have an excess of micropterous individuals while females have an excess of macropterous individuals.

Across both diet quality treatments, however, the nature of the trade-off between male reproduction and dispersal does not vary. Neither the frequency of occurrence of the wing morphs nor the relative differences in reproductive investment between macropterous and micropterous males differed between the diet treatments. Using multiple regression techniques based on selection analysis [58], we also assessed if the relationships between reproductive effort and a number of other important male and female characteristics differed for the two wing morph types or the two diet treatments. The relationships between the important life-history traits did not change between the micropterous and macropterous individuals or between the high and low quality diet treatments. For males, the relationships between nuptial gift size, body size and attractiveness remained the same as males who provided nuptial gifts for the greatest duration were both the largest and most attractive. Similarly for females there was no difference between diet treatments or wing morph in the relationship between predicted fecundity, body size and egg size as females with the highest predicted fecundity also lived the longest.

Together, our results suggest that the phenotypic correlations between investment in dispersal, reproduction, and morphology are consistent across populations of *Pteronemobius sp.* that have experienced different diets, even though the mean trait values for investment in each of these components have changed. Similar results have been found in *Gryllus firmus* whereby the relative difference between the two morphs in terms of male calling effort remains the same even during food limitation [59]. Importantly in *Gryllus firmus*, the potential differences between macropterous and micropterous individuals are not due to differences in the acquisition of resources. For example, Zera and Brink [56] demonstrated that there is no difference in the assimilation of lipid, carbohydrate or protein between the adult morphs. These patterns are consistent with quantitative genetic models of how relationships between life-history traits should evolve between populations where the environment and patterns of selection differ [32]. After short term or weak selection we should expect that a change in the magnitude or strength of a trade-off (the slope of a trade-off function) will be less likely than a shift in the mean trait values of a trade-off (the intercept). By manipulating the nutritional environment that populations experienced, we have documented a shift in average trait values but not the relationships between traits in a way that is consistent with the predictions of short term or weak selection. Dissecting how much of the differences between the populations are due to the effects of dietary intake versus genetic divergence will shed light on the important role of resource acquisition in the evolution of nuptial feeding mating systems and the adaptive plasticity of life-history trade-offs in general.
